# Herbivory and pollen limitation at the upper elevational range limit of two forest understory plants of eastern North America

**DOI:** 10.1002/ece3.3397

**Published:** 2017-12-12

**Authors:** Sébastien Rivest, Mark Vellend

**Affiliations:** ^1^ Département de Biologie Université de Sherbrooke Sherbrooke QC Canada

**Keywords:** climate change, elevational gradient, herbivory, pollen limitation, range limit

## Abstract

Studies of species' range limits focus most often on abiotic factors, although the strength of biotic interactions might also vary along environmental gradients and have strong demographic effects. For example, pollinator abundance might decrease at range limits due to harsh environmental conditions, and reduced plant density can reduce attractiveness to pollinators and increase or decrease herbivory. We tested for variation in the strength of pollen limitation and herbivory by ungulates along a gradient leading to the upper elevational range limits of *Trillium erectum* (Melanthiaceae) and *Erythronium americanum* (Liliaceae) in Mont Mégantic National Park, Québec, Canada. In *T. erectum,* pollen limitation was higher at the range limit, but seed set decreased only slightly with elevation and only in one of two years. In contrast, herbivory of *T. erectum* increased from <10% at low elevations to >60% at the upper elevational range limit. In *E. americanum*, we found no evidence of pollen limitation despite a significant decrease in seed set with elevation, and herbivory was low across the entire gradient. Overall, our results demonstrate the potential for relatively strong negative interactions (herbivory) and weak positive interactions (pollination) at plant range edges, although this was clearly species specific. To the extent that these interactions have important demographic consequences—highly likely for herbivory on *Trillium*, based on previous studies—such interactions might play a role in determining plant species' range limits along putatively climatic gradients.

## 1 INTRODUCTION

Understanding the factors limiting species distributions is a goal of increasing importance in ecology, as anthropogenic climate change is expected to induce widespread range shifts (Parmesan, 2006). Abiotic factors, particularly climate, have been extensively studied in this context (Sexton, McIntyre, Angert, & Rice, 2009). However, although theory suggests that biotic factors can also contribute to determining range limits (Case & Taper, 2000; Hochberg & Ives, 1999; Holt & Barfield, 2009), relatively few empirical studies have addressed this possibility (Sexton et al., 2009).

Plants are influenced by other species both negatively, via antagonistic interactions with competitors, herbivores, or pathogens, and positively, via mutualistic interactions with pollinators, seed dispersers, or mycorrhizal fungi. Plant–animal interactions such as pollination and herbivory have been repeatedly demonstrated to affect individual plant fitness and population growth (Maron, Baer, & Angert, 2014), but rarely in the context of range limits. Herbivores can have strong negative effects on plant growth, reproduction, and sometimes survival (Maron & Crone, 2006). Herbivory on roots and shoots can influence survival and growth via reduction in a plant's ability to acquire resources (Crawley, 2009), while herbivory on seeds and flowers (e.g., by rodents and insects) can influence plant abundance by reducing sexual reproduction (Maron & Crone, 2006). Compared to mammalian herbivores, insect herbivores tend to be more specialized, feeding on specific plant organs (Levin et al., 2009).

A majority of flowering plants rely on animal pollination vectors for reproduction (Ollerton, Winfree, & Tarrant, 2011) and insufficient pollen quantity or quality can reduce seed production and therefore the fitness of individual plants, a phenomenon known as pollen limitation (Ashman et al., 2004; Knight et al., 2005; Maron et al., 2014). Pollen limitation appears to be quite common: in an analysis of 482 studies, Knight et al. (2005) found that 63% of plant species suffered pollen limitation at certain times or localities. If population growth is sensitive to seed production, pollen limitation can negatively affect population growth rate, potentially leading to local extinction (Ashman et al., 2004). Of the few studies to date that have examined the effect of pollen limitation on population growth rate, some have found a negative effect (Kelly, Ladley, & Robertson, 2007; Parker, 1997; Price, Campbell, Waser, & Brody, 2008). A key first step in determining whether pollination limitation and/or herbivory can contribute to defining species' range limits (i.e., whether they prevent positive population growth) is to ask whether their strength increases as one approaches the range limit.

Plant–animal interactions are affected by environmental context, such that we might expect the strength of interactions to vary along environmental gradients (Hillyer & Silman, 2010; Louthan, Doak, & Angert, 2015; Straka & Starzomski, 2015; Totland, 2001). For example, pollinator abundance and activity can be limited by the same stressful abiotic conditions that limit plant populations (e.g., cold, drought, and nutrient scarcity). Thus, we might expect pollinator visitation to be reduced at plant range edges that are determined by abiotic stress (HilleRisLambers, Harsch, Ettinger, Ford, & Theobald, 2013; Moeller, Geber, Eckhart, & Tiffin, 2012). Reduced pollination might also occur via an indirect effect of the abiotic environment. If reduced environmental quality near the range edge reduces plant population size and density (Hardie & Hutchings, 2010; Kawecki, 2008), pollinators might be less attracted to forage in such plant populations (Dafni, Lehrer, & Kevan, 1997; Grindeland, Sletvold, & Ims, 2005; Karron, Thumser, Tucker, & Hessenauer, 1995; Pettersson & Sjodin, 2000). In either case, if plant population growth is sensitive to seed set, exacerbated pollen limitation might contribute to defining a plant species' range limit (Ashman et al., 2004). Of the few empirical studies conducted on this question to date, two found an increase in pollen limitation along aridity gradients (Chalcoff, Aizen, & Ezcurra, 2012; Moeller et al., 2012), one found an increase along an elevational gradient (Theobald, Gabrielyan, & HilleRisLambers, 2016), and three studies found no geographical trend (Busch, 2005; Hargreaves, Weiner, & Eckert, 2015; Stone & Jenkins, 2008).

Low plant density (e.g., at a range edge) might also result in either decreased (Fagan et al., 2005; Gunton & Kunin, 2007) or increased herbivory (Edwards, 1985; Kéry, Matthies, & Fischer, 2001). The Janzen–Connell hypothesis predicts that plants in low‐density patches benefit from release from enemy pressure (Connell, 1971; Janzen, 1970), while Root (1973) proposed that plants in denser or bigger patches might be more attractive to herbivores. Other models predict the opposite: a resource dilution effect at high densities, leading to greater herbivory per plant in smaller or less‐dense patches of plants (Otway, Hector, & Lawton, 2005). Many studies have also reported spatial variation in the magnitude of herbivory along gradients of elevation, sunlight, and disturbance (Maron & Crone, 2006). Among the few studies conducted to date on range limits specifically, some have found increases (Bruelheide & Scheidel, 1999; Galen, 1990) while others have found decreases (Alexander, Price, Houser, Finch, & Tourtellot, 2007; Urli, Brown, Perez, Chagnon, & Vellend, 2016) in herbivory toward plant species' range limits. New studies are clearly needed to permit generalizations and analyses of the contexts under which the strength of plant–animal interactions might vary along gradients leading up to species' range limits.

**Figure 1:**
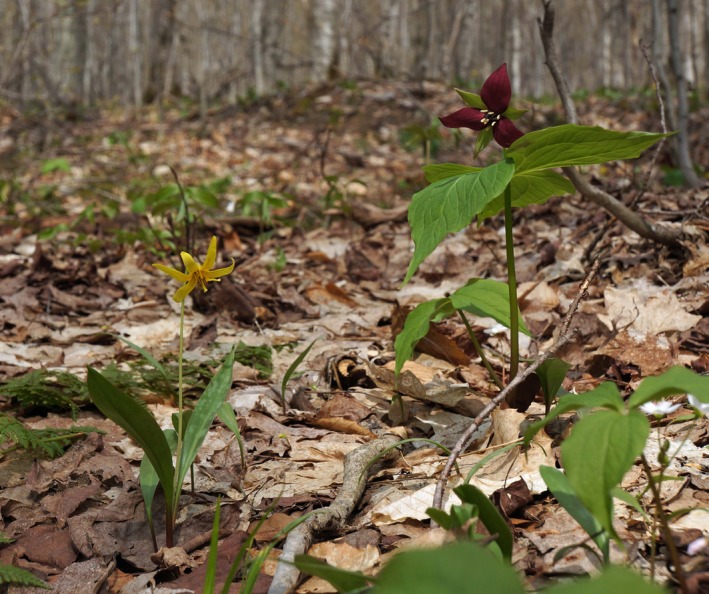
Flowering individuals of our two study species, *Erythronium americanum* (yellow flower) and *Trillium erectum* (red flower) at our study site on 18 May 2017

Here, we present observational and experimental data examining pollen limitation and herbivory on an elevational gradient in southern Québec, Canada, along which the upper range limits of our two focal forest understory plants (see Figure 1), *Erythronium americanum* Ker Gawl. (Liliaceae) and *Trillium erectum* L. (Melanthiaceae), are defined. First, we measured reproductive success in plants of both species along the elevational gradient, either with experimental pollen supplementation or not, to test the predictions that (1) plant reproductive success is lower and (2) pollen limitation is stronger at the upper elevational range limit than in populations at lower elevations. Previous studies indicate a greater capacity for self‐pollination in *T. erectum* (Broyles, Sherman‐Broyles, & Rogati, 1997; Irwin, 2000; Sage et al., 2001) than in *E. americanum* (Harder, Thomson, Cruzan, & Unnasch, 1985), such that we expected stronger pollen limitation for *E. americanum*. Second, by monitoring individual plants of both species, we tested whether herbivore damage by ungulates (white‐tailed deer and/or moose) varied along the elevational gradient. Given the theoretical possibility of either increases or decreases in herbivory at range edges, we had no *a priori* prediction concerning the direction of relationship between herbivory and elevation. However, *Trillium* species are strongly preferred food plants of white‐tailed deer (Anderson, 1994; Augustine, 1997; Rouleau, Crete, & Ouellet, 2002), such that we predicted greater herbivory on *T. erectum* than on *E. americanum*.

## 2 MATERIAL AND METHODS

### 2.1 Study species and plots

*Erythronium americanum* (Liliaceae) and *Trillium erectum* (Melanthiaceae) are perennial herbs native to the understory of the deciduous forests of eastern North America (Figure 1). Sexually reproductive individuals of *E. americanum* consist of a corm producing two basal leaves and a single nodding flower. For *T. erectum*, they are composed of one or occasionally 2–3 stems bearing a whorl of three leaves and a terminal flower originating from a tuber‐like rhizome. Individuals of both species flower early in the spring, producing one flower per stalk. Therefore, the number of seeds per fruit generally corresponds to the number of seeds per plant in a given year. *Erythronium americanum* flowers are pollinated primarily by Hymenoptera and Coleoptera (Bernhardt, 1977), while those of *T. erectum* produce a fetid odor that principally attracts dipterans (Irwin, 2000). The two species are considered partially self‐incompatible (Harder et al., 1985; Irwin, 2000; Sage et al., 2001).

Population growth of long‐lived perennials such as *E. americanum* and *T. erectum* is generally most sensitive to demographic transitions involving survival or growth of adults (Franco & Silvertown, 2004). For *T. grandiflorum*, a relative of *T. erectum* with near identical life history, herbivory by ungulates (involving removal of all leaf and reproductive tissue) has been demonstrated to have a strong negative impact on population growth by increasing the probability of regressing in size over time and decreasing the probability of future flowering (Knight, 2004).

**Figure 2:**
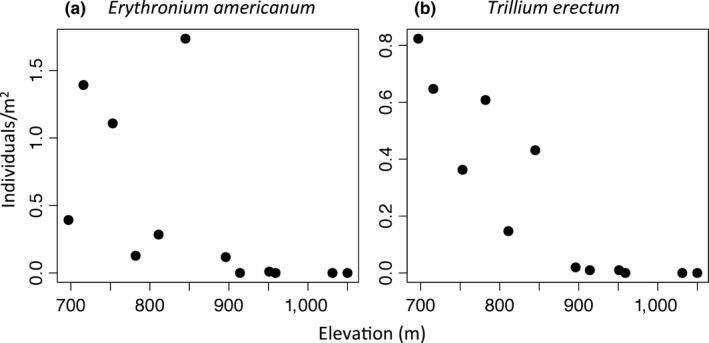
Elevational variation in the density of flowering individuals of *Erythronium americanum* (a) and *Trillium erectum* (b) on the eastern flank of Mont‐Saint‐Joseph. Data were taken during summer 2017 in the same 12 plots where plant phenology was studied by Lajoie and Vellend (2015). Each plot is 104 m^2^, and plots are arranged along two elevational transects adjacent to those used for the herbivory and pollen limitation experiment

Data were collected during the summers of 2015 and 2016 at Mont Mégantic National Park, located in southern Québec, Canada (45°26′51′'N, 71°06′52′'W), on the northern edge of the Appalachian mountain range. This protected area covers 55 km^2^, with an elevational gradient extending from ~500 m a.s.l. to 1105 m. Both focal species are abundant at lower elevations, but with few individuals found >900 m (occasional plants can be found up to ~960 m) (Figure 2). The vegetation varies from temperate deciduous forest, dominated by sugar maple, at low elevations (below ~800 m) to boreal forest, dominated by balsam fir and red spruce, at higher elevations (Lajoie & Vellend, 2015; Savage & Vellend, 2015). Along the gradient, mean annual temperature shifts from 3.6°C to 0.4°C, reducing the length of the growing season from approximately 100–80 days (Parc National du Mont Mégantic 2016).

We established two elevational transects on the east‐facing slope of Mont‐St‐Joseph, on the eastern edge of the park. Transects were separated by approximately 1 km, each consisting of one plot each at low (700 m elevation), mid (800 m), and high elevations (900 m, near the range limit) (Appendix S1, see Supplemental Data with the online version of this article). The three plots were located in deciduous, mixed, and boreal forest, respectively. Given the low abundance of *E. americanum* and *T. erectum* at high elevation, high‐elevation plots had to be larger (~100 × 40 m) than those at mid and low elevation (80 × 20 m) to obtain a sufficient sample size. Low‐elevation plots do not represent the lower elevational range limits; both species are found at lower elevations, beyond the plateau (400–500 m elevation) that surrounds the park, but abundance of both species is as high at the low‐elevation plots at Mont Mégantic as it is anywhere else in the broader region (personal observation).

### 2.2 Reproduction along the elevational gradient

To quantify natural patterns of seed set in 2015, we harvested 29–34 ripe fruits per plot, per species. In 2016, we used fruits collected from the control plants in the pollen limitation experiment (see below). In 2015, at low and mid elevations, we randomly selected subplots of 2 × 2 m in which fruits were collected from all sexually reproductive plants; subplots were added until a total of ~30 fruits had been collected (transect 1: 20 and 18 subplots at low and mid elevations, respectively; transect 2: 20 and 21 subplots at low and mid elevations). At high elevation, the entire plot was searched and fruits were collected until a sample of ~30 had been reached. In order to allow for subplot to be included as a random factor in analyses (see below), subplots at high elevation were defined as areas of less than 5 × 5 m containing at least two individuals (five subplots for transect 1, four subplots for transect 2).

Fruit collection occurred just prior to ripening, when filled seeds were clearly discernible but before fruits had fallen from the plant. At low, mid, and high elevations, respectively, fruits were collected on days of the year 164–165, 174–175, and 182–183 (*E. americanum*) and 216–217, 222–223, and 230–231 (*T. erectum*). For each fruit, we counted the number of fully developed (filled) seeds, aborted seeds, and undeveloped ovules under a bifocal microscope. Developed seeds are large and plump, while aborted seeds are smaller, wrinkled, and brown, and undeveloped ovules are very small, discolored, and wrinkled. Fruits that never developed were recorded as having zero seeds (reproductive success = 0; it was impossible to count ovules in these fruits). For each individual, leaf lengths and widths were measured to provide an estimate of plant size (leaf area), for use as a covariate in statistical analyses. For *E. americanum,* these measurements were taken on the two leaves, which typically differ in size. For *T. erectum*, leaf size is strongly correlated across leaves within a plant (r = 0.99), so measurements were taken on only one haphazardly selected leaf per plant (see Appendix S2).

### 2.3 Pollen limitation experiment

In 2016, we performed a pollen supplementation experiment to test whether seed set was limited by access to outcross pollen and if the magnitude of pollen limitation was greatest at the range limit. For each species at each plot, between 75 and 92 plants were selected in a stratified random way (see below) before flower opening. Plant damage (largely due to herbivory) reduced final sample sizes per plot to 30–90 (mean = 58) for *T. erectum* and 62–87 (mean = 75) for *E. americanum*. Pairs of plants were identified based on physical proximity as well as similarity in size and phenology. In each pair, each individual was randomly assigned to the open pollination or pollen supplementation treatments. At low‐ and mid‐elevation plots, plant pairs were evenly distributed across randomly selected subplots of 5 × 5 m with a maximum of 10 individuals (five pairs) per subplot (transect 1: 16 subplots at low elevation, 15 at mid elevation; transect 2: 18 subplots at low elevation, 19 at mid elevation). Given the low density of flowering individuals at high‐elevation plots, all plants in these plots were included in the experiment, with subplots defined as described above (20 subplots on transect 1, seven subplots on transect 2).

Plants in the pollen supplementation group were manually pollinated once, using paintbrushes, within 2 days of the onset of stigma receptivity (determined visually). As the onset of stigma receptivity did not occur on the same day for all plants in a given plot, pollen supplementation at a given plot was performed over a period of 4–5 days. Pollen was applied until the stigma was completely covered. On each day of pollen supplementation, we collected recently dehisced anthers from at least five donor plants located at least 20 m outside the plots and used the mixed pollen for the supplementation treatment. We measured leaf length and width on each plant as in 2015 and harvested all fruits on approximately the same dates as in the 2015 observational study of reproductive success.

### 2.4 Herbivory across the elevational gradient

In 2015, we qualitatively observed an increase in deer or moose herbivory (both species are present at the site) with elevation for *T. erectum*. Ungulates remove all leaf and reproductive tissue in *Trillium*, leaving behind a straight cut on the remaining stem as evidence of herbivory (Knight, 2004), while for sexually reproducing *Erythronium* plants, ungulate herbivory is identified by a cut on the flower stalk and the loss of the upper part of each leaf. In 2016, we quantified the presence or absence of ungulate herbivory on each plant of both species in the pollen experiment by monitoring plants every 2–3 days during the flowering period (from day of the year 127 to 160) and every week during fruit development (from day of the year 160–183 for *E. americanum* and to 230 for *T. erectum,* following Knight, 2004). We also monitored evidence of insect damage on plants, but the proportion of plants affected was negligible (<4% at all elevations); these data were not included in our analyses.

### 2.5 Statistical analyses

For both species, we calculated an index of leaf area from their leaf length and width (Appendix S2), for use as a covariate in the following analyses. To analyse natural variation in the number of seeds per fruit (absolute seed set) and seeds per ovule (relative seed set: number of seeds divided by the total number of ovules) along the elevational gradient, we used generalized linear mixed models (GLMMs) with subplot as a random effect. Elevation was treated as a categorical variable (low, mid, and high) in all analyses. Separate analyses were conducted for each species. Seeds per fruit models assumed a negative binomial distribution of residuals, given the high dispersion parameter values in each model; models for seeds per ovule assumed a binomial distribution. The effect of transect and the transect × elevation interaction were modeled as fixed effects, given the low number of transects (2). We used leaf area as a covariate for *T. erectum*, but not for *E. americanum*, given a large number of missing values in 2015 (leaves had often withered before fruit collection). We also tested for variation in leaf area across elevations, using plants observed in both 2015 and 2016 in general linear mixed models including subplot as random effect and transect as a fixed effect.

We used GLMMs to analyse, for both species independently, the effect of pollen supplementation and its interaction with elevation on seeds per fruit, assuming a negative binomial distribution. For seeds per ovule, we assumed a binomial distribution. We used pairs nested within subplots as random effects, the leaf area estimate as a covariate, and transect and the transect × elevation interaction as fixed effects. Pollen limitation was inferred from the treatment effect in the statistical model, and variation of the level of pollen limitation with elevation was assessed by the interaction between pollination treatment and elevation.

To test if herbivory varied with elevation, we used GLMMs with a Bernoulli distribution, with pairs nested in subplots as random effects, and transects and the transect × elevation interaction as fixed effects. For *E. americanum*, complete separation of the data, owing to the absence of herbivory at high‐elevation plots, required the addition of informative priors in order to estimate the fixed effects (a prior variance of 9 for each fixed‐effect parameter) (Abrahantes & Aerts, 2012). When there was a significant effect of elevation (*p *< .05), we tested for differences among elevations using Tukey post hoc tests.

For each model, we used a backward model selection approach using log‐likelihood ratio tests. We always retained variables relating directly to our hypotheses (elevation, treatment) as well as estimated plant size, given its clear ecological interpretation. Random factors, the transect and year effects (when relevant), and associated interactions were dropped from models if they were not significant (*p* > .05); when an interaction was significant, associated main effects were also retained. Analyses were conducted in R version 3.1.3 (R Development Core Team, 2015). We used the lme4 package for fitting mixed‐effects models (Bates, Machler, Bolker, & Walker, 2015), the MASS package for negative binomial models (Venables & Ripley, 2002), the blme package for mixed‐effects models with informative priors of the fixed‐effect parameters (Chung, Rabe‐Hesketh, Dorie, Gelman, & Liu, 2013), and the lsmean package for post hoc tests (Lenth, 2016).

## 3 RESULTS

### 3.1 Natural patterns of seed set

**Figure 3:**
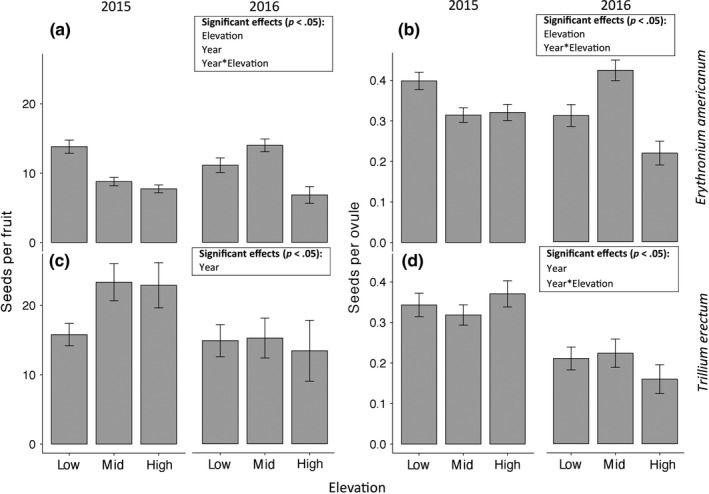
Elevational variation in seeds per fruit (absolute seed set, a, c) and seeds per ovule (relative seed set, b, d) for *Erythronium americanum* (a, b) and *Trillium erectum* (c, d) in 2015 and 2016. Significant predictors (not including transect effects) are shown in each panel, with statistical details presented in Table 1. Graphs show means ± 1 SE of raw data across all individual plants

Elevational patterns in reproductive success differed between species (Figure 3; Table 1). In *Erythronium americanum*, elevation had a significant effect on number of seeds per fruit (Figure 3a, absolute seed set) and number of seeds per ovule (Figure 3b, relative seed set), both of which were lower at mid and/or high elevations compared to low elevation (Figure 3a,b; Table 1). The number of seeds per fruit (but not seeds per ovule) was significantly higher in 2015 than in 2016. There was also a significant interaction between year and elevation for both measures of reproductive success in *E. americanum*; at mid elevations, both seeds per fruit and seeds per ovule were relatively low in 2015 and high in 2016. For *Trillium erectum*, there was no effect of elevation on seeds per fruit or seeds per ovule (Figure 3c,d; Table 1). The number of seeds per fruit and per ovule was significantly higher in 2015 compared to 2016. The number of seeds per ovule in *T. erectum* at high elevation was relatively high in 2015 and relatively low in 2016 (significant interaction between year and elevation). For both species, leaf area was a strong predictor of seeds per fruit (larger plants producing more seeds; for all tests: *p* ≤ .0007, Tables 1 and 2), but not of seeds per ovule (for all tests: *p* > .05, Tables 1 and 2). Average plant size varied significantly among elevations for *E. americanum* in both years and for *T. erectum* in 2016, but for both species in 2016, the largest plants were actually found at the highest elevations (Appendix S4).

**Table 1:** Generalized linear mixed model results for the effects of elevation on seeds per fruit (absolute seed set, assuming negative binomial distributions) and seeds per ovule (relative seed set, assuming binomial distributions) for *Erythronium americanum* and *Trillium erectum* in 2015 and 2016 (see Appendix S3 for details of the random effects). Effects were tested with Wald chi‐square‐tests

Model	Effect	χ^2^	*df*	*p*
Seeds per fruit, *E. americanum*	Intercept	705.15	1	**<.0001**
	Elevation	27.32	2	**<.0001**
	Year	5.22	1	**.022**
	Year*Elevation	26.00	2	**<.0001**
	Transect	2.87	1	.09
	Transect*Elevation	7.03	2	**.03**
*T. erectum*	Intercept	93.21	1	**<.0001**
	Elevation	3.03	2	.22
	Leaf area	429.68	1	**<.0001**
	Year	14.84	1	**.0001**
	Transect	13.54	1	**.0002**
Seeds per ovule, *E. americanum*	Intercept	194.54	1	**<.0001**
	Elevation	29.85	2	**<.0001**
	Year	0.18	1	.67
	Year*Elevation	17.47	2	**.0001**
	Transect	4.52	1	.033
	Transect*Elevation	16.91	2	**.0002**
*T. erectum*	Intercept	116.49	1	**<.0001**
	Elevation	0.82	2	.66
	Leaf area	4.62	1	**.031**
	Year	3.74	1	.053
	Year*Elevation	16.26	2	**.0003**

Bold indicates *p* < 0.05.

**Table 2:** Generalized linear mixed model results for the effects of elevation, pollination treatment (control or pollen supplementation), and their interaction on seeds per fruit (absolute seed set) and seeds per ovule (relative seed set) for *Erythronium americanum* and *Trillium erectum* in 2016 (assuming negative binomial distributions, see Appendix S3 for details of the random effects). Effects were tested with Wald chi‐square‐tests

Model	Effect	χ^2^	*df*	*p*
Seeds per fruit, *E. americanum*	Intercept	265.36	1	**<.0001**
	Elevation	30.92	2	**<.0001**
	Pollination treatment	2.16	1	.14
	Pollination treatment*Elevation	0.76	2	.69
	Leaf area	11.76	1	**.0006**
*T. erectum*	Intercept	0.43	1	.51
	Elevation	3.28	2	.19
	Pollination treatment	2.15	1	.14
	Pollination treatment*Elevation	12.45	2	**.002**
	Leaf area	184.46	1	**<.0001**
	Transect	10.63	1	**.0011**
Seeds per ovule, *E. americanum*	Intercept	129.82	1	**<.0001**
	Elevation	6.59	2	.037
	Pollination treatment	0.12	1	.73
	Pollination treatment*Elevation	0.74	2	.69
	Leaf area	0.30	1	.59
	Transect	2.85	1	.09
	Transect*Elevation	11.26	2	**.0036**
*T. erectum*	Intercept	125.22	1	**<.0001**
	Elevation	17.84	2	**.0001**
	Pollination treatment	0.13	1	.72
	Pollination treatment*Elevation	6.88	2	**.032**
	Leaf area	0.95	1	.33

### 3.2 Pollen limitation

**Figure 4:**
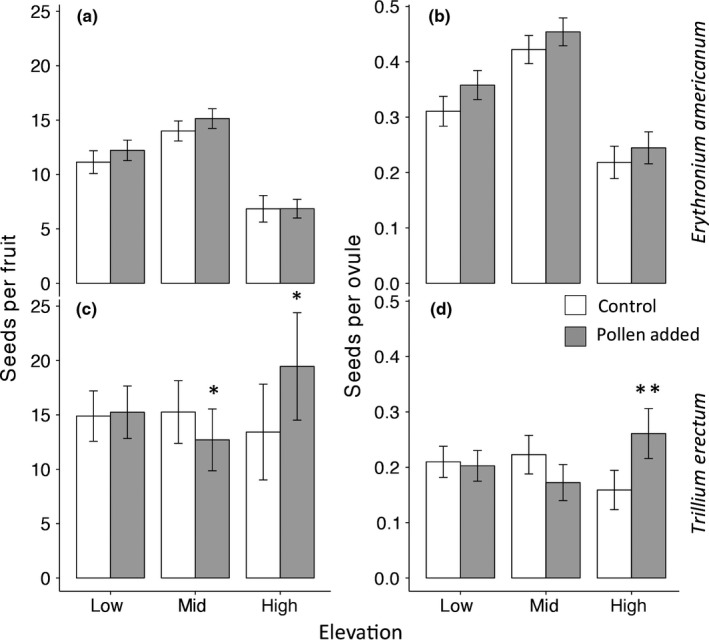
The effect of pollen supplementation on seeds per fruit (absolute seed set, a, c) and seeds per ovule (relative seed set, b, d) along the elevational gradient for *Erythronium americanum* (a, b) and *Trillium erectum* (c, d) in 2016. White bars indicate control flowers while gray bars indicate pollen‐supplemented flowers. Asterisks above bars indicate a significantly larger difference between pollination treatments (i.e., greater or lower pollen limitation) compared to the low‐elevation group (*p < .05, **p < .01). Graphs show means ± 1 SE of raw data across all individual plants

Patterns of pollen limitation were different between the two species. Overall, seed set in *E. americanum* was not pollen limited, regardless of elevation (i.e., no significant effect of treatment, either alone or in interaction with other factors; Figure 4a,b; Table 2). In *T. erectum*, there was no significant pollen limitation overall, but there was a significant elevation × pollination treatment interaction for both seeds per fruit and seeds per ovule, with pollen limitation greater at high than low elevation (Figure 4c,d; Table 2). At high elevation (i.e., the range edge), pollen supplementation increased seeds per fruit by 30% (mean ± 1 SE across all individual plants = 14.17 ± 4.62 for the control group and 20.06 ± 5.06 for the pollen supplementation group) and seeds per ovule by 38% (mean ± 1 SE across all individual plants = 0.17 ± 0.04 for the control group and 0.27 ± 0.05 for the pollen supplementation group).

### 3.3 Herbivory

**Figure 5:**
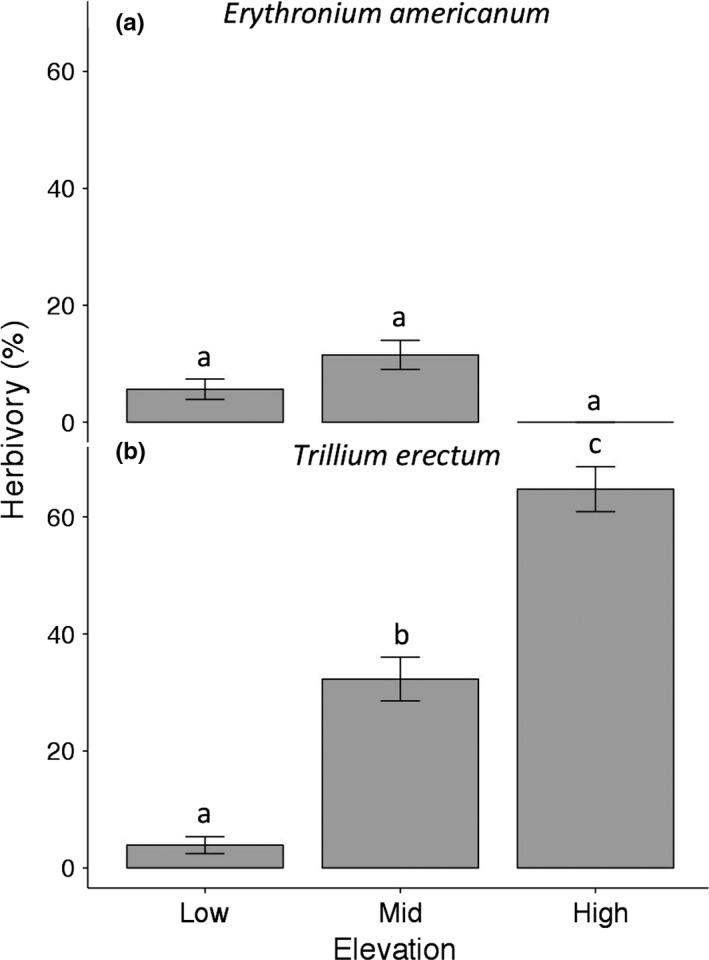
Elevational variation in percentage herbivory of *Erythronium americanum* (a) and *Trillium erectum* (b). Different letters above bars indicate statistically significant differences between groups (α = 0.05, Tukey post hoc tests). Graphs show means ± 1 SE of raw data across all individual plants

For both species, herbivory was <10% at low elevation (Figure 5). Herbivory of sexually reproducing *E. americanum* plants remained low at mid and high elevations as well, with no significant effect of elevation (Figure 5a). For *T. erectum*, herbivory showed a significant and marked increase toward the range limit, from <10% at low elevation to >60% at high elevation (Figure 5b; Table 3), consistent with our qualitative observations in 2015.

**Table 3:** Generalized linear mixed model results for the effects of elevation on herbivory for *Erythronium americanum* and *Trillium erectum* in 2016 (assuming Bernoulli distributions, see Appendix S3 for details of the random effects). Effects were tested with Wald chi‐square‐tests

Model	Effect	χ^2^	*df*	*p*
*E. americanum*	Intercept	3.25	1	.07
	Elevation	0.006	2	.99
*T. erectum*	Intercept	29.53	1	**<.0001**
	Elevation	28.59	2	**<.0001**
	Transect	1.23	1	.27
	Transect*Elevation	18.09	2	**.0001**

### 3.4 Comparison of transects

Results on the two transects were largely consistent for seed set, pollen limitation, and herbivory (Appendix S5). Although the transect × elevation interaction was significant in several models (see Tables 1, 2, 3), in most cases, the effect of elevation was in the same direction on both transects but of different magnitude, or the response variables were different largely at mid elevation (and only in 2016), rather than at low or high elevation (Appendix S5). One exception was for seeds per fruit in *Trillium*, which appeared to increase with elevation on one transect and to decrease with elevation on the other (Appendix S5), although this has no effect on our conclusions described in the Discussion.

## 4 DISCUSSION

Our study suggests that the strength of biotic interactions can differ significantly between plant populations at the edge of a species' range and populations in the core of the species' range, albeit in a species‐specific way. While both herbivory and pollen limitation were strongest at the upper elevational range limit of *Trillium erectum,* no trends with elevation were observed for *Erythronium americanum*. Although the potential importance of biotic interactions in defining range limits has been noted in the literature (HilleRisLambers et al., 2013; Soberon, 2007), to date few empirical studies have directly quantified the importance of multiple interactions along gradients leading up to range edges.

Our most striking result was the marked increase in herbivory toward the upper elevational range limit of *T. erectum*, from <10% at low elevation to more than 60% at the range limit (Figure 5b). The herbivory we observed was characteristic of ungulate browsing (jagged and torn straight cut on the remaining stem opposed to a clean 45° cut for snowshoe hares, *Lepus americanus* (Williams, Mosbacher, & Moriarity, 2000)) and was most likely due to white‐tailed deer (*Odocoileus virginianus*), which occur in both deciduous and boreal forests at Mont Mégantic. The creation of anthropogenic landscapes (a mix of fields and forests at different successional stages) has resulted in high densities of white‐tailed deer throughout eastern North America, with major impacts on plant communities (Russell, Zippin, & Fowler, 2001). Many studies have shown *Trillium* to be a preferred food plant of white‐tailed deer (Anderson, 1994; Augustine, 1997; Rouleau et al., 2002). Moose (*Alces americana*) are also present in boreal forests at Mont Mégantic and elsewhere (Martin, Zim, & Nelson, 1961; Pastor, Naiman, Dewey, & McInnes, 1988), but to our knowledge, there are no reports of moose consumption of *Trillium*.

Previous studies indicate that high deer herbivory, which we found to be greatest at the range edge, likely has an impact on *Trillium* population dynamics. First, browsing on *Trillium* plants causes 100% defoliation and a near‐complete loss of annual seed production (a few seeds might survive gut passage and be dispersed by deer, Vellend, Myers, Gardescu, & Marks, 2003). In addition, *Trillium* species do not reproduce clonally (rhizomes are only a few cm long), and there is no aboveground regrowth within the growing season (Augustine & Frelich, 1998; Knight, 2003). Deer herbivory, therefore, limits the storage of carbohydrate in the rhizome, typically resulting in smaller plants the following year (Knight, 2003; Lubbers & Lechowicz, 1989). Kalisz, Spigler, and Horvitz (2014) demonstrated that exclusion of overabundant deer resulted in a significant increase in *T. erectum* population growth rate and size. Also, for a closely related species with a very similar life history, *T. grandiflorum*, Knight, Caswell, and Kalisz (2009) demonstrated that deer browsing of reproductive plants at a rate of >15% led to population decline, with population growth especially sensitive to demographic transitions involving the largest plants (i.e., those targeted by deer). In our study, range‐edge populations of *T. erectum* suffered browsing of more than 60% in 2016, with similarly high herbivory observed qualitatively in 2015. This level of herbivory almost certainly has a strong negative impact on population growth via an increased probability of size regression or mortality and might limit upward range expansion, thus helping to define the range limit. However, in the absence of detailed demographic data, we cannot infer whether these populations are currently declining. White‐tailed deer densities have increased in recent decades throughout much of their range (Garrott, White, & Callie, 1993), so it is possible that this level of herbivory at the range limit is a recent phenomenon, which would explain why peripheral populations still have many reproductive individuals. These issues are clearly in need of further study.

Interestingly, in other studies in which increased herbivory was observed at a range limit, for example by slugs (Bruelheide & Scheidel, 1999) as well as ungulates and aphids (Galen, 1990), it was at the lower elevational limit, where less stressful conditions are thought to promote a greater abundance of herbivores (Menge & Sutherland, 1987). In our study, it is possible that deer are more abundant at higher elevations, but this seems unlikely given that boreal forest is not typically the preferred habitat for deer (Hewitt, 2011). We hypothesize instead that deer are showing increased selectivity for *Trillium* plants in the boreal forest. *Trillium erectum* density is reduced close to the upper elevational range limit at our study site (Figure 2), and there is some evidence of increased proportional herbivory by white‐tailed deer with decreasing density in *Trillium* populations (Augustine & Frelich, 1998). Studies are underway to test these alternative hypotheses.

We also found greater pollen limitation near the range limit, again only for *T. erectum*. For this species, pollen supplementation did not affect reproductive success at lower elevations, while at the range limit it increased seed production by more than 30% (Figure 4c,d). On the surface, this result suggests that pollen limitation might contribute to determining the upper elevational range limit of *T. erectum*. However, compared to low‐elevation populations, reproductive success in unmanipulated plants was only slightly reduced at the range limit in 2016 (the year we assessed pollen limitation) and was not significantly different in 2015. Moreover, the 30% increase in reproductive success due to pollen supplementation at the range limit actually increased reproductive success beyond that observed in plants in either treatment at lower elevations (Figure 4c,d). In addition, the pollen limitation observed in 2016 might not be representative of other years, such as 2015, during which reproductive success was not decreased at the range limit. Several other studies have found pollen limitation only in some years for a given population (Kameyama et al., 2015; Stone & Jenkins, 2008; Theobald et al., 2016), possibly owing to interannual variation in pollinator activity or abundance or to variation in resource availability. Finally, in a study of *T. grandiflorum*, Knight (2004) demonstrated that a level of pollen limitation similar to that observed in our study had a minimal impact on population growth, particularly compared to herbivory.

In short, our results for *T. erectum* do not indicate that pollen limitation is likely to be a major factor in determining the upper elevational range limit. That said, pollen limitation often has its strongest effects on population growth in establishing populations (Knight et al., 2005), such that it might influence the rate of range expansion, even if the effect on current population growth at the range edge is minimal. The increase in pollen limitation we observed at the range limit could be due to a decrease in pollinator abundance (HilleRisLambers et al., 2013; Moeller et al., 2012), or decreased attraction of pollinators resulting from reduced plant size or density (Elliott & Irwin, 2009; Grindeland et al., 2005). We did not find reduced plant size closer to the range limit (Appendix S4C, D), but *T. erectum* density does decrease with increasing elevation at our study site (see Figure 2). High herbivory at the range limit might also contribute to reduced flower density.

In sexually reproducing *E. americanum* plants, we observed no trends with elevation for pollen limitation or herbivory, although reproductive success was reduced at the range limit. Givnish (1990) and La Rocca, Pupillo, Puppi, and Rascio (2014) have proposed that the leaf mottling of *E. americanum* and other *Erythronium* species might serve as camouflage from herbivory, particularly by dichromats (species with only two types of color receptor in their eyes) such as deer. Thus, *E. americanum* might largely avoid herbivory by deer, particularly when present at low density. *Erythronium* leaves are also present over a shorter period of time in the spring than *Trillium* leaves (Lapointe, 2001), allowing less time for browsing. Consistent with this interpretation, Rouleau et al. (2002) observed 3.8 times greater abundance of *Trillium* than *E. americanum* in the rumen of white‐tailed deer, despite both species being of comparable abundance at their study site.

In terms of pollen limitation, the difference we observed between species contradicted our prediction that *E. americanum* would be more responsive to pollen supplementation. Differences in reproductive strategy are thus not likely the cause of the difference in pollen limitation we observed between the two species*. Trillium erectum*, which showed some evidence of pollen limitation in our experiment, is considered more self‐compatible (Harder et al., 1985; Irwin, 2000; Sage et al., 2001), which is predicted to result in lower susceptibility to pollen limitation (Lloyd, 1992). However, the two species are visited by different sets of pollinators. *Erythronium americanum* flowers are visited principally by Hymenoptera and Coleoptera (Bernhardt, 1977) while *T. erectum* flowers are visited by Diptera (Irwin, 2000). At our study site, bumblebees (*Bombus* spp.) are often seen visiting *E. americanum*. Bumblebees have been demonstrated to be effective pollinators of *E. americanum* and other *Erythronium* species with similar morphology (Theobald et al., 2016; Thomson, 1986; Thomson & Thomson, 1989), even in cold environments (Arroyo, Kalin, Primack, & Armesto, 1982; Bingham & Orthner, 1998). Thus, more reliable pollination by bumblebees than by *T. erectum* pollinators with increasing elevation might explain the difference between our two study species. Additionally, Theobald et al. (2016) observed pollen limitation at the upper elevation range limit of a different *Erythronium* species (*E. montanum*), but only in years when visitation by bumblebees was low. Reliable pollination by bumblebees at our study site might also explain the difference between our results and those of Theobald et al. (2016).

Despite not showing evidence of pollen limitation, reproductive success decreased more consistently with elevation for *E. americanum* than for *T. erectum* (Figure 3). Limitation of reproductive success can be caused by insufficient pollen receipt and/or insufficient resources available for fruit production (Ashman et al., 2004; Burd, 1994; Knight et al., 2005). In the case of *E. americanum*, we expect that abiotic environmental factors (e.g., climate, soil, or light) are responsible for reduced reproductive success at high elevations. A plant's capacity to allocate resources to reproduction is generally strongly correlated with plant size (Silvertown & Charlesworth, 2009), and we observed a significant relationship between leaf area and seeds per fruit in our study species, as well. However, plant size was lower at the range limit in only one of the two years, with the opposite pattern in the other year (Appendix S4).

In a broader context, models predicting the effect of climate change on species distributions are most often based only on abiotic factors (Gilman, Urban, Tewksbury, Gilchrist, & Holt, 2010; VanDerWal, Shoo, Johnson, & Williams, 2009). Our study, along with other recent studies (e.g., Moeller et al., 2012; Chalcoff et al., 2012; Brown and Vellend 2014, Urli et al., 2016; Stanton‐Geddes, Tiffin, & Shaw, 2012), suggests that biotic interactions require greater consideration in order to provide reliable predictions of the consequences of climate warming for species distributions, although results are clearly species specific. In our case, biotic factors, particularly herbivory, are potentially constraining population growth at the leading edge of *T. erectum*'s distribution and therefore may limit its capacity for migration under climate change (HilleRisLambers et al., 2013). Long‐term demographic studies are needed to further test this hypothesis. To the extent that white‐tailed deer prefer deciduous forest plants to boreal forest plants more generally (e.g., due to adaptations to nutrient rich vs. poor soils), our finding might have broader implications for plant migration at the deciduous‐boreal forest ecotone.

## CONFLICT OF INTEREST

None declared.

## AUTHOR CONTRIBUTIONS

SR and MV designed the study and interpreted the results collectively. SR conducted the field work and laboratory measurements and wrote the manuscript, which was revised and edited by MV.
