# Supplementary material for: Herbivory and pollen limitation at the upper elevational range limit of two forest understory plants of eastern North America
Source: Ecol Evol. 2017 Dec 12;8(2):892–903. doi: 10.1002/ece3.3397 (PMC5773324; doi:10.1002/ece3.3397)
Supplement: Supplementary file 1 [file ECE3-8-892-s001.docx]

**APPENDIX S1**

**
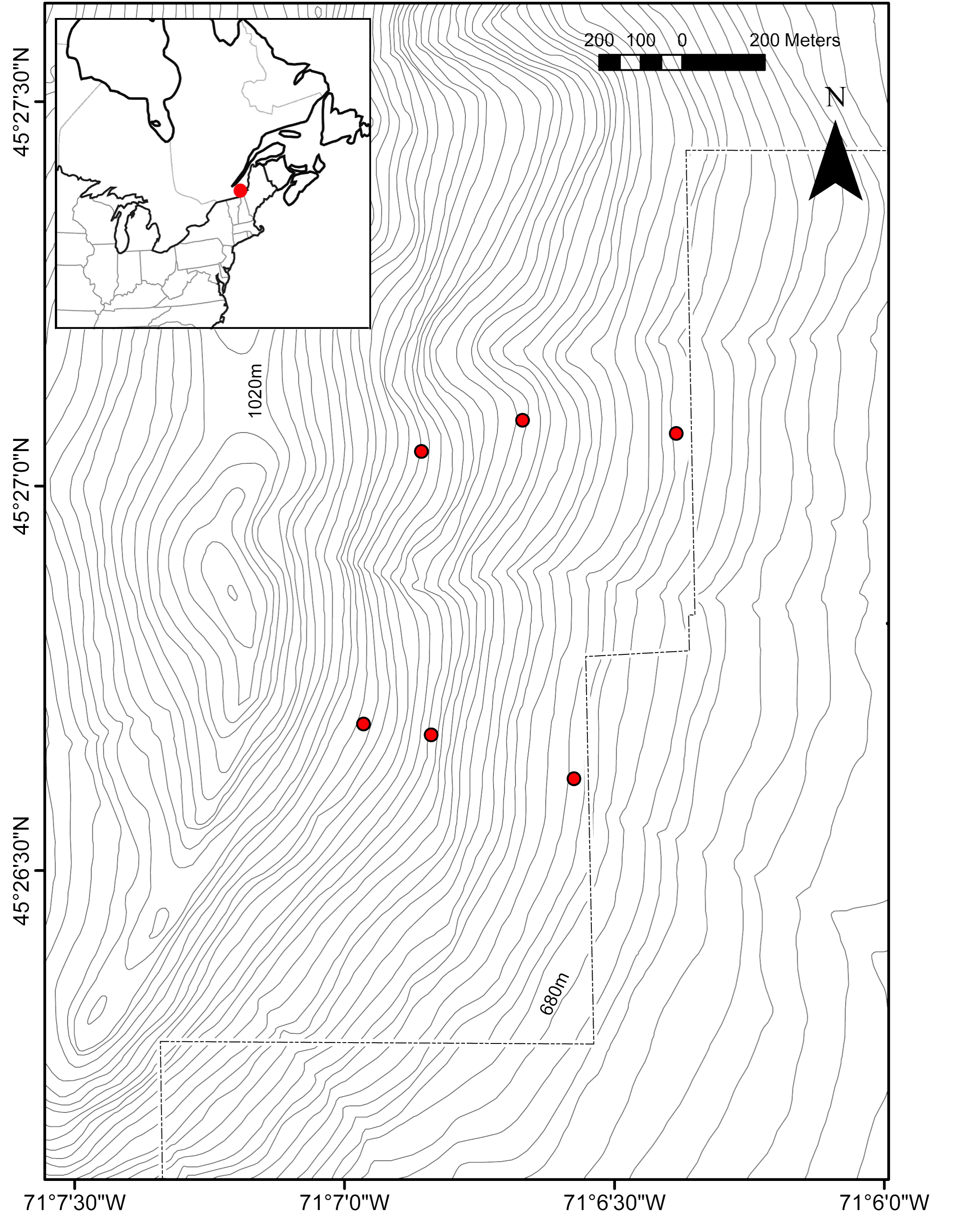
**

**Figure 1.** Map of experimental sites on the eastern flank of Mont-Saint-Joseph, from low (right) to high (left) elevation, along two transects. The dotted line shows the boundary of Parc national du Mont Mégantic, with the park being to the west of the line. Contour lines are at 10 m intervals.
