# Supplementary material for: Herbivory and pollen limitation at the upper elevational range limit of two forest understory plants of eastern North America
Source: Ecol Evol. 2017 Dec 12;8(2):892–903. doi: 10.1002/ece3.3397 (PMC5773324; doi:10.1002/ece3.3397)
Supplement: Supplementary file 2 [file ECE3-8-892-s002.docx]

**APPENDIX S2**

As an index of plant size, for *Erythronium americanum*, we calculated the product of length and width of each leaf (reproductive individuals have two leaves). For *Trillium erectum* we calculated the product of the length and width of one of the three leaves (the leaves are arranged in a whorl and are approximately of the same size). For both species, we estimated total leaf area by first collecting (outside the experimental plots) 50 sexually reproductive plants covering a wide range of sizes, and measuring the length and width of the leaves. Each of these plants was digitally scanned, and their total leaf area estimated using ImageJ (Schneider, Rasband, and Eliceiri, 2012)^[[1]](#footnote-1)^. We then used general linear models to predict total leaf area with the product (P) of leaf length and width of the measured leaves, assuming a zero y-intercept. We used the resulting regression equations with estimated coefficients (B_1_ = 0.53 for *T. erectum*; B_1_ = 0.64 and B_2_ = 0.62 for *E. americanum*) to calculate total leaf area (R^2^ = 0.98 for *T. erectum* and 0.99 for *E. americanum*): *T. erectum* leaf area = B_1_ x 3P, *E. americanum* $leaf area=B_{1} x P$_leaf1_ + B_2_  x P_leaf2_.

1. Schneider, C.A., Rasband, W.S. and Eliceiri, K.W. 2012 NIH Image to ImageJ: 25 Years of Image Analysis. Nature Methods, 9:671-675. [↑](#footnote-ref-1)
