# Supplementary material for: Herbivory and pollen limitation at the upper elevational range limit of two forest understory plants of eastern North America
Source: Ecol Evol. 2017 Dec 12;8(2):892–903. doi: 10.1002/ece3.3397 (PMC5773324; doi:10.1002/ece3.3397)
Supplement: Supplementary file 3 [file ECE3-8-892-s003.docx]

**APPENDIX S3**

**Table 1.** Variance and standard deviation of the random effects included in the generalized linear mixed models from Table 1 for the effects of elevation on seeds per fruit (absolute seed set, assuming negative binomial distributions) and seeds per ovule (relative seed set, assuming binomial distributions) for *Erythronium americanum* and *Trillium erectum*.

| **Model** | **Random effect** | **Variance** | **Standard deviation** |
| --- | --- | --- | --- |
| Seeds per fruit,  *E. americanum* | Subplot | 0.2319 | 0.4816 |
| *T. erectum* | Subplot | 8.608 | 2.934 |
| Seeds per ovule,  *E. americanum* | Subplot | 0.03608 | 0.1899 |
| *T. erectum* | Subplot | 0.1769 | 0.4206 |

**Table 2.** Variance and standard deviation of the random effects included in the generalized linear mixed models from Table 2 for the effects of elevation, pollination treatment (control or pollen supplementation) and their interaction on seeds per fruit (absolute seed set) and seeds per ovule (relative seed set) for *Erythronium americanum* and *Trillium erectum* (assuming negative binomial distributions).

| **Model** | **Random effect** | **Variance** | **Standard deviation** |
| --- | --- | --- | --- |
| Seeds per fruit,  *E. americanum* | Subplot | 0.1976 | 0.4445 |
|  | Pair | 0.5925 | 0.7697 |
| *T. erectum* | Subplot | 4.809 | 2.193 |
|  | Pair | 8.976 | 2.996 |
| Seeds per ovule,  *E. americanum* | Subplot | 0.02663 | 0.1632 |
|  | Pair | 0.02155 | 0.1468 |
| *T. erectum* | Pair | 0.1725 | 0.4154 |

**Table 3.** Variance and standard deviation of the random effects included in the generalized linear mixed model from Table 3 for the effects of elevation on herbivory for *Erythronium americanum* and *Trillium erectum* (assuming Bernoulli distributions).

| **Model** | **Random effect** | **Variance** | **Standard deviation** |
| --- | --- | --- | --- |
| *E. americanum* | Pair | 231.9 | 15.23 |
| *T. erectum* | Pair | 1.437 | 1.199 |
