# Supplementary material for: Herbivory and pollen limitation at the upper elevational range limit of two forest understory plants of eastern North America
Source: Ecol Evol. 2017 Dec 12;8(2):892–903. doi: 10.1002/ece3.3397 (PMC5773324; doi:10.1002/ece3.3397)
Supplement: Supplementary file 4 [file ECE3-8-892-s004.docx]

**APPENDIX S4**

**Figure 1.** Elevational variation in leaf area for *Erythronium americanum* (A. B) and *Trillium erectum* (C, D) in 2015 (A, C) and 2016 (B, D). For *Erythronium americanum* there was a significant effect of elevation on leaf area in 2015 and 2016. *Erythronium americanum* plants had reduced leaf area at high elevation compared to low elevation in 2015 (t = -2.41, P =0.021) but the opposite trend was observed in 2016 (t = 2.11, P = 0.037). For *Trillium erectum*, there was no significant effect of elevation on leaf area in 2015. In 2016, mid and high elevation plants had greater leaf area than low elevation plants (high elevation: t = 7.89, P < 0.0001; mid elevation: t = 7.17, P < 0.0001).
