# Supplementary material for: Herbivory and pollen limitation at the upper elevational range limit of two forest understory plants of eastern North America
Source: Ecol Evol. 2017 Dec 12;8(2):892–903. doi: 10.1002/ece3.3397 (PMC5773324; doi:10.1002/ece3.3397)
Supplement: Supplementary file 5 [file ECE3-8-892-s005.docx]

**APPENDIX S5**

**Figure 1.** Elevational variation in seeds per fruit (absolute seed set, A-D) and seeds per ovule (relative seed set, E-H) on each transect for *Erythronium americanum* (A, B, E, F) and *Trillium erectum* (C, D, G, H) in 2015 (A, C, E, G) and 2016 (B, D, F, H). Graphs show means and standard errors on raw data.

**Figure 2.** The effect of pollen supplementation on seeds per fruit (absolute seed set, A-D) and seeds per ovule (relative seed set, E-H) along the elevational transects A (A, C, E, G) and B (B, D, F, H) for *Erythronium americanum* (A, B, E, F) and *Trillium erectum* (C, D, G, H) in 2016. White bars indicate control flowers while grey bars indicate pollen-supplemented flowers. Graphs show means and standard errors on raw data.

**Figure 3.** Elevational variation in percentage herbivory of *Erythronium americanum* (A) and *Trillium erectum* (B) along each transect. Graphs show means and standard errors on raw data.
